# Preliminary Metabolomics Study Suggests Favorable Metabolic Changes in the Plasma of Breast Cancer Patients after Surgery and Adjuvant Treatment

**DOI:** 10.3390/biomedicines12102196

**Published:** 2024-09-26

**Authors:** Andrea Jiménez-Franco, Juan Manuel Jiménez-Aguilar, Marta Canela-Capdevila, Raquel García-Pablo, Helena Castañé, Cristian Martínez-Navidad, Pablo Araguas, Bárbara Malavé, Rocío Benavides-Villarreal, Johana C. Acosta, Alina Iuliana Onoiu, Navita Somaiah, Jordi Camps, Jorge Joven, Meritxell Arenas

**Affiliations:** 1 Unitat de Recerca Biomèdica, Hospital Universitari Sant Joan de Reus, Institut d’Investigació Sanitària Pere Virgili, Universitat Rovira i Virgili, Av. Dr. Josep Laporte 2, 43204 Reus, Spain; andrea.jimenez@urv.cat (A.J.-F.); juanmaaguilar106@gmail.com (J.M.J.-A.); marta.canela@iispv.cat (M.C.-C.); raquel.garciap@estudiants.urv.cat (R.G.-P.); helena.castane@iispv.cat (H.C.); cristian.martinez@iispv.cat (C.M.-N.); americadelrocio.benavides@salutsantjoan.cat (R.B.-V.); johana.acosta@salutsantjoan.cat (J.C.A.); alinaiuliana.onoiu@urv.cat (A.I.O.); meritxell.arenas@urv.cat (M.A.); 2 Department of Radiation Oncology, Hospital Universitari Sant Joan de Reus, Institut d’Investigació Sanitària Pere Virgili, Universitat Rovira i Virgili, Av. Dr. Josep Laporte 2, 43204 Reus, Spain; pablo.araguasmora@gmail.com (P.A.); barbaraantonia.malave@salutsantjoan.cat (B.M.); 3 The Royal Marsden NHS Foundation Trust and Division of Radiotherapy and Imaging, Institute of Cancer Research, 131-139 Dovehouse St, London SW3 6JZ, UK; navita.somaiah@icr.ac.uk

**Keywords:** breast cancer, chemotherapy, metabolism, metabolomics, radiotherapy

## Abstract

**Background/Objectives**: The management of early breast cancer (BC) includes surgery, followed by adjuvant radiotherapy, chemotherapy, hormone therapy, or immunotherapy. However, the influence of these interventions in metabolic reprogramming remains unknown. This study explored alterations in the plasma metabolome of BC patients following distinct treatments to deepen our understanding of BC pathophysiology, outcomes, and the identification of potential biomarkers. **Methods**: We included 52 women diagnosed with BC and candidates for surgery as primary oncological treatment. Blood samples were collected at diagnosis, two weeks post-surgery, and one month post-radiotherapy. Plasma samples from 49 healthy women served as controls. Targeted metabolomics assessed 74 metabolites spanning carbohydrates, amino acids, lipids, nucleotide pathways, energy metabolism, and xenobiotic biodegradation. **Results**: Before treatment, the BC patients exhibited notable changes in carbohydrate, nucleotide, lipid, and amino acid metabolism. We noticed a gradual restoration of specific metabolite levels (hypoxanthine, 3-phosphoglyceric acid, xylonic acid, and maltose) throughout different treatments, suggesting a normalization of the nucleotide and carbohydrate metabolic pathways. Moreover, we observed increased dodecanoic acid concentrations, a metabolite associated with cancer protection. These variations distinguished patients from controls with high specificity and sensitivity. **Conclusions**: Our preliminary study suggests that oncological treatments modify the metabolism of patients towards a favorable profile with a decrease in the pathways that favor cell proliferation and an increase in the levels of anticancer molecules. These findings emphasize the pivotal role of metabolomics in recognizing the biological pathways influenced by each cancer treatment and the resulting metabolic consequences. Furthermore, it aids in identifying potential biomarkers for disease onset and progression.

## 1. Introduction

Breast cancer (BC) is the most prevalent solid neoplasm and the primary cause of cancer-related death among females. In 2022, BC accounted for the second-highest incidence of all cancers globally, with approximately 2.3 million new cases, constituting 11.6% of all cancer types, leading to nearly 666,000 deaths [1].

BC is a complex and heterogeneous disease that requires personalized treatment tailored to the characteristics of each patient and the cancer stage, molecular subtype, and other aggressiveness factors [2]. The usual therapeutic modalities for BC include surgery, radiotherapy (RT), adjuvant chemotherapy, targeted therapy, hormonal therapy, and, increasingly, neoadjuvant therapy, which is used before surgery in selected cases to reduce tumor size and guide treatment decisions based on tumor response [3,4]. BC treatments aim to remove or destroy cancerous cells, prevent the spread of the disease, and improve overall survival and quality of life [5].

Recent studies have revealed the relationship between metabolism and oncogenesis [6,7], making it plausible to hypothesize that various metabolic alterations may be the cause or effect of the different efficacy in response to oncological treatments. Improved knowledge in this area would aid treatment planning and monitoring, enhancing our understanding of its effects on tumor cells.

Cancer cells display a unique and accelerated energy metabolism, allowing them to extract vital nutrients from an often nutrient-deprived environment. This metabolic adaptation supports their growth, proliferation, and viability [8]. The resulting metabolic changes give rise to characteristic phenotypes that can be leveraged to identify potential biomarkers crucial for diagnosing and evaluating treatment responses. Moreover, these insights can inform strategies for patient selection in clinical settings, contributing to more effective and personalized approaches to cancer treatment [9].

Metabolomics offers distinctive insights into disease pathogenicity by identifying disease-related determinants and treatment responses [9,10,11]. Analyzing the downstream molecular effects of treatment is crucial in discovering new therapeutic alternatives and candidate biomarkers for the diagnosis, prognosis, and monitoring of treatment responses. Furthermore, it allows for mapping biochemical pathways drugs target in cancer cells [12].

Our study is distinguished from previous research by its focus on the specific plasma metabolic alterations in early-stage BC before and after primary treatments such as surgery and RT. Through a detailed metabolomic analysis, we identified significant changes in specific metabolites. These metabolites may serve as putative biomarkers for evaluating treatment efficacy, potentially improving therapeutic planning for early-stage cancer patients. We employed advanced statistical methods such as multivariate analysis and pathway enrichment analysis to analyze the complex data. These methods allowed us to integrate the results and explore potential metabolic pathways and molecular targets with high precision and reliability.

## 2. Materials and Methods

The study involved 52 women diagnosed with BC between September 2020 and October 2021. The inclusion criteria were to be a woman ≥18 years old and to have an invasive cancer. We excluded patients with a history of previous oncological disease, BC metastatic stage, Paget’s nipple disease, vascular collagen disease, systemic lupus erythematosus, scleroderma, pregnancy, lactation, and metabolic or psychiatric diseases. We also excluded patients with a COVID-19-positive result by polymerase chain reaction or antigen test during the study period. Although neoadjuvant therapy has become common, its use depends on multiple factors, including molecular subtypes, tumor size, lymph nodes, and individual patient characteristics. Therefore, we have chosen to limit the heterogeneity of our patients, selecting only those who underwent surgery and subsequent therapies without prior neoadjuvant treatment.

All the patients underwent treatment with surgery and RT, often supplemented with adjuvant chemotherapy and hormonal therapy tailored to their molecular subtype, as well as other risk factors, overall patient health, and age. Of these, 40 patients underwent lumpectomy, while 12 opted for mastectomy. RT was administered to the breast or mastectomy site, with or without nodal irradiation. The radiation schedule was hypofractionated RT (40 Gy at 2.67 Gy/day, five days/week) to the breast or mastectomy site. Following whole-breast irradiation, 34 patients received an additional boost at the tumor bed (16 Gy at 2 Gy/day or 13.34 Gy at 2.67 Gy/day, five days/week) [13,14]. Blood samples were obtained at the BC diagnosis, two weeks after surgery, and one month after RT. Regrettably, the timing of the post-RT sample collection in our hospital coincided with a surge in COVID-19 admissions, limiting our ability to obtain samples to only 26 patients. Aliquots of whole blood from each patient were immediately processed for hematological analysis, and other aliquots were collected in tubes without added anticoagulant or in tubes with EDTA and centrifuged at 2500× *g* to obtain serum and plasma. They were then stored at −80 °C until batched analyses. As a control group, we employed plasma samples from 49 women participating in a population-based study conducted in our area. They had no clinical or analytical evidence of cancer, renal failure, liver disease, or neurological disorders [15].

All the participants signed a written informed consent according to the Helsinki Declaration. The study was approved by the Ethics Committee of our Institution.

Semi-quantitative metabolomics was employed to determine the plasma concentration of 74 metabolites involved in carbohydrates, amino acids, lipids, cofactors, vitamins, nucleotide pathways, energy metabolism, and xenobiotic biodegradation [16]. The determination was carried out using a combination of gas chromatography (GC), electron impact ionization (EII), mass spectrometry (MS), and a high-resolution time-of-flight (QTOF) analyzer (GC-EI-QTOF-MS) to provide an accurate identification of the components present in the sample even among those with similar masses [17].

A 50 μL aliquot of plasma was deposited into a 1.5 mL Eppendorf tube and amalgamated with 200 μL of an 8:2 (*v*/*v*) methanol–water solution, which included the internal standards. The specific internal standards used are detailed in Table S1. Following the mixture, the samples were vortexed and centrifuged at 15,000 rpm and 4 °C for 5 min. Subsequently, supernatants (200 μL) were decanted into a fresh tube and subjected to evaporation in a SpeedVac vacuum concentrator (Thermo Fisher Scientific, Waltham, MA, USA) at 45 °C. The samples were then reconstituted using 30 μL of methoxyamine and placed in an incubator at 37 °C for 90 min. The final step involved silylating the samples with 45 μL of N-Methyl-N-trimethylsilyl-trifluoroacetamide supplemented with 1% trimethylchlorosilane at room temperature for 60 min. The chromatographic separation was performed in a 7890A gas chromatograph paired with a 7200-quadruple time-of-flight mass spectrometer equipped with an electron impact source (Agilent Technologies, Santa Clara, CA, USA). Moreover, the system was fitted with a 7693 autosampler module and a J&W Scientific HP-5MS column (30 ms 0.25 mm, 0.25 μm) from Agilent Technologies. The analytes’ identification and semi-quantification were achieved using relative units (RU), determined by the ratio of the compound area to the internal standard area. More specifically, ions were selected and used for quantitation based on their impact electron spectra (70 eV) and the primary specific ions recorded in the Fiehn-pct-2013 spectral library.

The serum concentration of the antioxidant enzyme paraoxonase-1 (PON1) was analyzed with the Human PON1 ELISA kit from Elabscience^®^ (Houston, TX, USA). Serum PON1 activity was analyzed by the rate of the hydrolysis of phenylacetate at 280 nm, in a 9 mM Tris-HCl buffer, pH 8.0, and supplemented with 0.9 mM CaCl2, as previously described [18]. The plasma concentrations of chemokine (C-C motif) ligand 2 (CCL2) and interleukin-10 (IL-10) were quantified with ABTS ELISA Development kits (Peprotech, London, UK). Serum glucose, creatinine, and C-reactive protein concentrations, lipid profile, and hepatic enzymes were determined via standard methods using a COBAS^®^ 8000 automated analyzer (Roche Diagnostics, Basel, Switzerland). The total blood count was determined in a Sysmex XN-1000 automated hematology analyzer (Sysmex, Kobe, Japan).

We performed group comparisons using the Mann–Whitney U test for quantitative variables and the χ^2^ square test for categorical variables. Statistical significance was set at *p* < 0.05. Quantitative variables are reported as median (interquartile range), and qualitative variables are presented as frequency (percentage).

Partial least squares-discriminant analysis (PLS-DA), Variable Importance Projection (VIP) score, volcano plots, receiver operating characteristics (ROC) curves, and enrichment analysis were made with MetaboAnalyst 5.0 (www.metaboanalyst.ca, accessed on 20 August 2024). Data visualizations, such as boxplots, network correlation analysis, bubble plots, and multiple regression analyses were crafted using RStudio 4.4.1. All the R packages were the latest versions available on CRAN (https://cran.r-project.org/, accessed on 20 August 2024) as of 14 June 2024. The boxplots were designed to illustrate variations in metabolite concentrations across different groups. This involved the strategic use of R packages such as ggplot2 for generating the plots, ggsignif for adding statistical significance annotations, gridExtra for arranging multiple plots, ggpubr for enhancing publication quality, and patchwork for combining plots into a cohesive layout. Network correlation analysis was conducted to explore relationships between metabolites, biochemical variables, and cytokines. This analysis employed igraph for constructing and visualizing network graphs, Hmisc for calculating correlation matrices, qgraph for creating clear network visualizations, and dplyr for data manipulation and preparation. Additionally, bubble plots were generated to visualize concentration changes among metabolites based on their species. The packages used were ggplot2, dplyr, and ggrepel. The TableOne package was employed to compare the clinical characteristics between the BC patients and controls. To analyze and present the metabolite concentrations, the dplyr and tidyr packages were used to calculate medians and ranges.

To assess the influence of age, dyslipidemia, hypertension, and smoking status on metabolite concentrations, we employed multiple linear regression models using the lm function in R. For each metabolite, we included age, dyslipidemia, hypertension, and smoking status as predictors in the model. We compared models with and without each variable to evaluate its individual effect. The results indicated that none of the potential confounding variables significantly influenced metabolite concentrations; therefore, no further adjustments were necessary.

A sample size calculation using standard statistical methods [19] determined that 45 subjects in the control group and 25 subjects in the post-treatment BC group would be required to detect a minimum difference of 0.7 RU in the mean concentrations of dodecanoic acid, with an alpha risk of 0.05 and a beta risk of 0.2.

## 3. Results

### 3.1. Clinical and Analytical Characteristics of BC Patients and the Control Group

We observed significant differences in age, dyslipidemia, arterial hypertension, and smoking habits between the two cohorts. Most patients were postmenopausal, had a history of motherhood, and reported a family history of cancer. Additionally, the BC patients showed a more atherogenic biochemical profile than the healthy individuals. Specifically, they had significantly elevated serum glucose, total cholesterol, very low-density lipoprotein cholesterol, triglycerides, γ-glutamyl transferase, and IL-10 levels, alongside decreased serum PON1 activity, compared to the control group (Table 1).

Tumor classification based on the TNM staging system revealed predominant categories of T1, N0, and M0. Additionally, a prevailing histological grade II was noted. Histopathological analyses revealed that carcinoma ductal and luminal B was the most prevalent tumor phenotype. Most tumors tested positive for estrogen and progesterone receptors and displayed a Ki67 antigen proliferation frequency ranging from 12% to 30% (Table 2).

### 3.2. Metabolite Baseline Levels Discriminate between BC Patients and the Control Group

**Figure 1:**
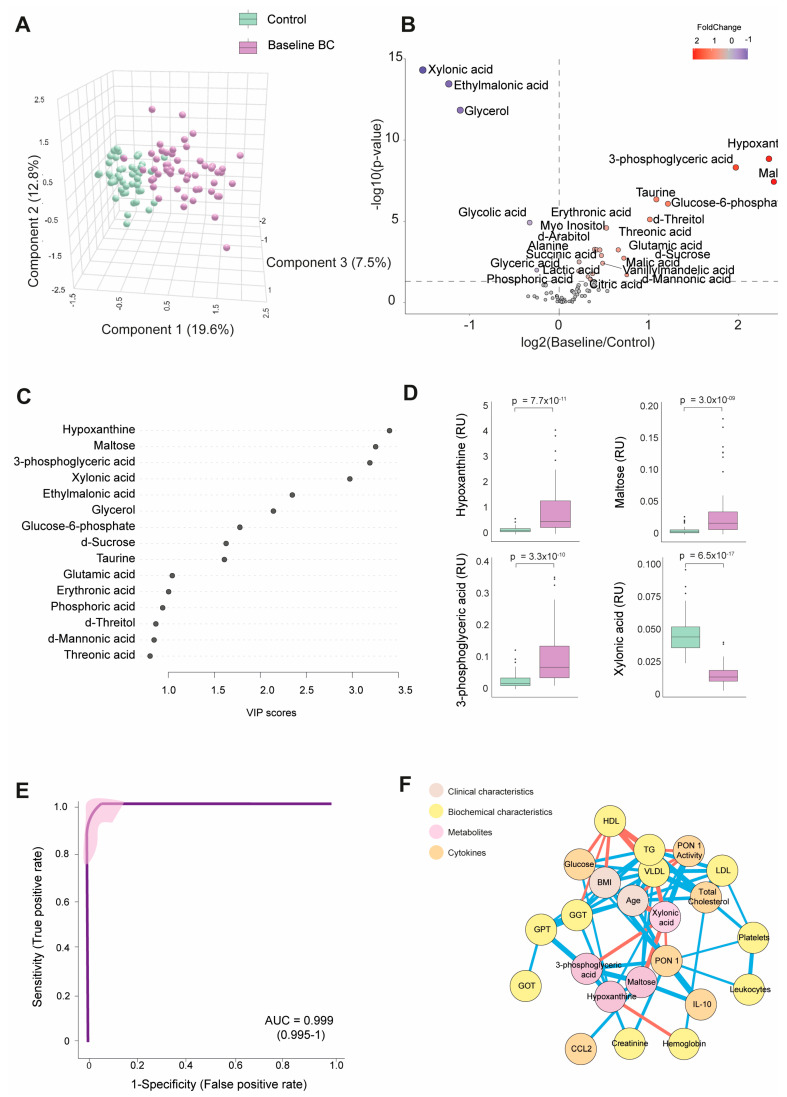
Discrimination of the breast cancer (BC) patients from the control group based on baseline plasma metabolite levels. (**A**) The BC patients displayed a distinct metabolic profile compared to the control group. (**B**) The majority of metabolites were elevated in the BC patients. (**C**) Variable Importance in Projection (VIP) underscores the critical roles of hypoxanthine, maltose, 3-phosphoglyceric acid, and xylonic acid in distinguishing between the two groups. (**D**) Hypoxanthine, maltose, and 3-phosphoglyceric acid levels were higher in the BC patients, while xylonic acid levels were lower than in the control group. (**E**) The receiver operating characteristic (ROC) curve and confusion matrix illustrate the effectiveness of hypoxanthine, maltose, 3-phosphoglyceric acid, and xylonic acid as discriminative markers between the BC patients and the control group. (**F**) Network correlation analysis identified positive (blue lines) and negative (red lines) correlations between the selected metabolites and various variables, with line thickness representing the interaction strength. Statistical significance was assessed using the Mann–Whitney U test. AUC: area under the curve; CCL2: chemokine (C-C motif) ligand 2; GGT: γ-glutamyl transferase; GOT: glutamate-oxaloacetate transaminase; GPT: glutamate-pyruvate transaminase; HDL: high-density lipoprotein; IL: interleukin; LDL: low-density lipoprotein; PON1: paroxonase-1; RU: relative units; TG: triglycerides; VLDL: very low-density lipoprotein.

Table S2 shows the numerical values of the examined metabolite concentrations. The BC patients had 25 higher and 8 lower metabolite concentrations than the control group. As depicted in Figure 1A, PLS-DA effectively segregated the metabolic profiles of both subject groups. The volcano plot shows that most metabolites were elevated at baseline, with notable increases in hypoxanthine, maltose, and 3-phosphoglyceric acid. In contrast, xylonic acid was decreased. (Figure 1B).

Through a random forest analysis, hypoxanthine, maltose, 3-phosphoglyceric acid, and xylonic acid emerged as the metabolites with the highest discriminatory potential between BC patients and healthy subjects. Specifically, the first three metabolites displayed heightened levels in the BC patients. At the same time, xylonic acid exhibited a lower concentration, as portrayed in Figure 1C,D. The ROC curve constructed using the combination of these four parameters yielded an area under the curve (AUC) value of 0.999 (Figure 1E). The correlation network analysis shows how these four metabolites correlated with biochemical variables and cytokines. Hypoxanthine correlated negatively with hemoglobin and positively with creatinine, GGT, glucose, and PON1 activity. Notably, the nodes for glucose and PON1 activity were distant. 3-phosphoglyceric acid correlated negatively with xylonic acid and positively with GPT, PON1, and maltose. Maltose correlated negatively with xylonic acid and positively with IL-10, 3-phosphoglyceric acid, and very low-density lipoprotein cholesterol (VLDL). Xylonic acid correlated negatively with maltose, 3-phosphoglyceric acid, age, and VLDL but positively with PON1 activity (Figure 1F).

The analysis of the four key metabolites (hypoxanthine, maltose, 3-phosphoglyceric acid, and xylonic acid) about tumor-related variables did not reveal any significant differences. No substantial variations were observed in the metabolite concentrations based on the clinical and tumor characteristics assessed. Differences in age, sex, and comorbidities between the BC patients and controls did not affect plasma metabolite concentrations (Figure S1).

### 3.3. Changes in the Metabolic Signature of BC Patients Post-Surgery and Post-RT

**Figure 2:**
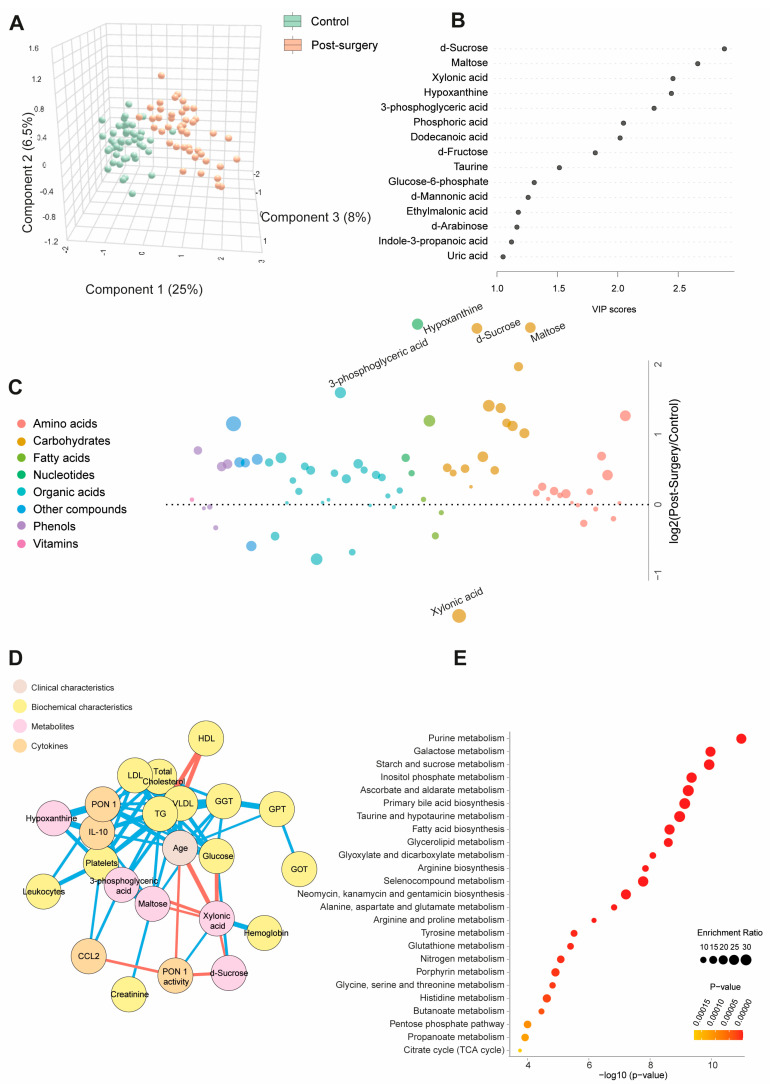
Changes in plasma metabolite levels in the breast cancer (BC) patients post-surgery compared to the control group. (**A**) The comparison of metabolic profiles between the post-surgery patients and controls revealed distinct differences in metabolite concentrations, underscoring significant variations in their metabolic states. (**B**,**C**) The random forest analysis identified d-sucrose, maltose, xylonic acid, and hypoxanthine as the most influential metabolites. (**D**) The network correlation analysis showed both positive (blue lines) and negative (red lines) correlations between the selected metabolites and various variables, with line thickness representing the strength of the interactions. (**E**) The enrichment analysis identified significantly altered pathways, notably purine and galactose metabolism, which exhibited marked changes in response to the condition under study. Statistical significance was assessed using the Mann–Whitney U test. CCL2: chemokine (C-C motif) ligand 2; GGT: γ-glutamyl transferase; GOT: glutamate-oxaloacetate transaminase; GPT: glutamate pyruvate transaminase; HDL: high-density lipoprotein; IL: interleukin; LDL: low-density lipoprotein; PON1: paroxonase-1; TG: triglycerides; VLDL: very low-density lipoprotein.

Table S3 and Figure 2A show quantitative data on the metabolite concentrations in the BC patients post-surgery compared to the control group. The BC patients exhibited 45 higher and 8 lower metabolite concentrations post-surgery. The PLS-DA analysis revealed a distinct metabolic profile between the two groups. The random forest analysis identified sucrose, maltose, hypoxanthine (elevated), and xylonic acid (reduced) as the metabolites with the highest discriminatory power for distinguishing BC patients from healthy subjects. The bubble plot demonstrated that most metabolites increased post-surgery (Figure 2B,C). The network correlation analysis showed that d-sucrose negatively correlated with PON1 activity and xylonic acid while positively correlating with glucose. Maltose negatively correlated with xylonic acid but positively with GGT, VLDL, triglycerides, and IL-10. Xylonic acid negatively correlated with d-sucrose, maltose, 3-phosphoglyceric acid, age, and glucose but positively with hemoglobin and PON1 activity. Hypoxanthine positively correlated with PON1, IL-10, and platelets (Figure 2D). The enrichment analysis highlighted significant alterations in metabolic pathways, notably purine metabolism and galactose metabolism, providing critical insights into the metabolic shifts associated with breast cancer (Figure 2E). Differences in age, sex, and comorbidities between the BC patients and controls did not affect the plasma d-sucrose, hypoxanthine, maltose, 3-phosphoglyceric acid, and xylonic acid concentrations (Figure S2).

**Figure 3:**
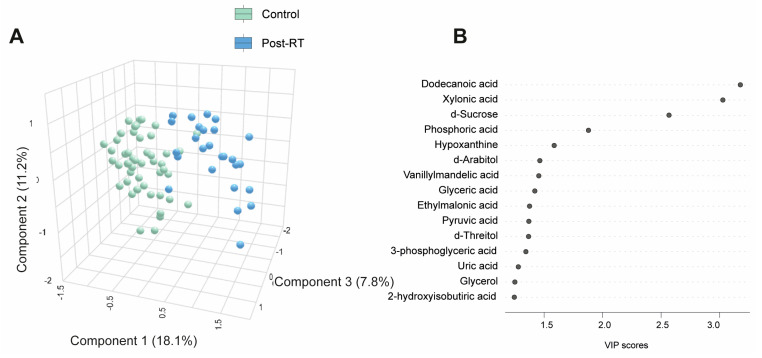

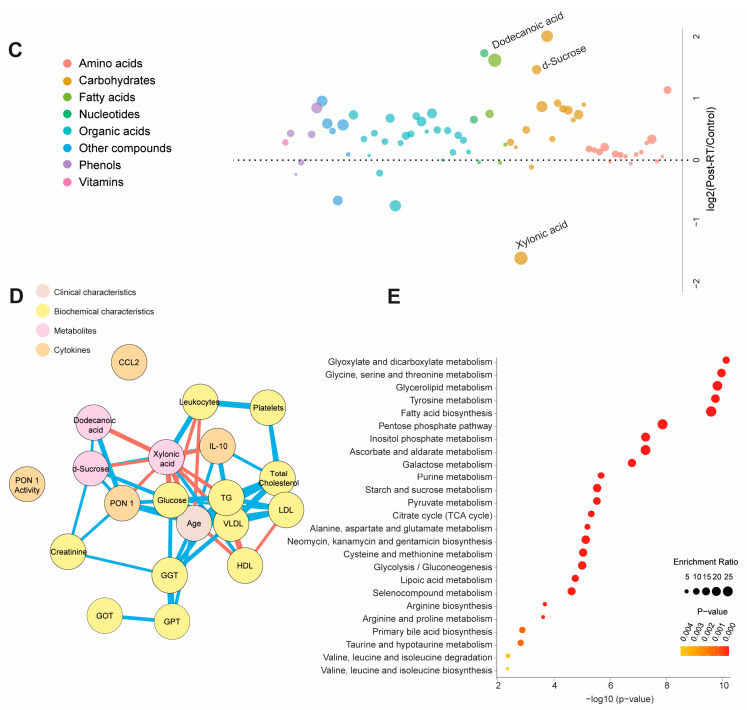
Alterations in plasma metabolite levels in breast cancer (BC) patients post-radiotherapy (RT) compared to the control group. (**A**) PLS-DA analysis demonstrated a clear separation between the two groups, indicating distinct metabolic differences. (**B**,**C**) The random forest analysis identified dodecanoic acid, xylonic acid, and d-sucrose as the most prominent metabolites. (**D**) The network correlation analysis revealed positive (blue lines) and negative (red lines) correlations between the selected metabolites and various variables, with line thickness indicating the strength of these interactions. (**E**) Enrichment analysis uncovered significant alterations in several metabolic pathways, including glycolate metabolism, dicarboxylate metabolism, and the metabolism of glycine, serine, and threonine. These pathways exhibited notable changes in the post-radiotherapy group compared to the controls. Statistical significance was assessed using the Mann–Whitney U test. CCL2: chemokine (C-C motif) ligand 2; GGT: γ-glutamyl transferase; GOT: glutamate-oxaloacetate transaminase; GPT: glutamate-pyruvate transaminase; HDL: high-density lipoprotein; IL: interleukin; LDL: low-density lipoprotein; PON1: paroxonase-1; TG: triglycerides; VLDL: very low-density lipoprotein.

Table S4 and Figure 3A present quantitative data on the metabolite concentrations in the BC patients post-radiotherapy (post-RT) compared to the control group. We observed 41 higher and 5 lower metabolite concentrations in patients. The PLS-DA analysis revealed significant differences in the metabolic profiles between the control and post-RT groups, underscoring distinct metabolic alterations associated with RT (Figure 3A). The random forest analysis identified dodecanoic acid, sucrose (elevated), and xylonic acid (reduced) as the metabolites with the highest discriminatory potential for distinguishing BC patients from healthy subjects (Figure 3B,C). The network correlation analysis showed dodecanoic acid negatively correlated with xylonic acid but positively correlated with d-sucrose and PON1. Xylonic acid negatively correlated with dodecanoic acid, d-sucrose, PON1, glucose, age, LDL, triglycerides, and IL-10 while positively correlated with leukocytes. d-sucrose negatively correlated with xylonic acid and positively with dodecanoic acid, PON1, glucose, and IL-10 (Figure 3D). The enrichment analysis highlighted the most significantly altered pathways, including glyoxylate and dicarboxylate metabolism, as well as glycine, serine, and threonine metabolism (Figure 3E). Additionally, we investigated whether there were post-RT differences in metabolite concentrations between the patients who received adjuvant chemotherapy and those who did not. The differences were slight, but we observed higher levels in five metabolites and lower levels in three metabolites (Table S5). Differences in age, sex, and comorbidities between the BC patients and controls did not affect the plasma d-sucrose, dodecanoic acid, and xylonic acid concentrations (Figure S3).

### 3.4. Main Metabolic Changes after Treatments

**Figure 4:**
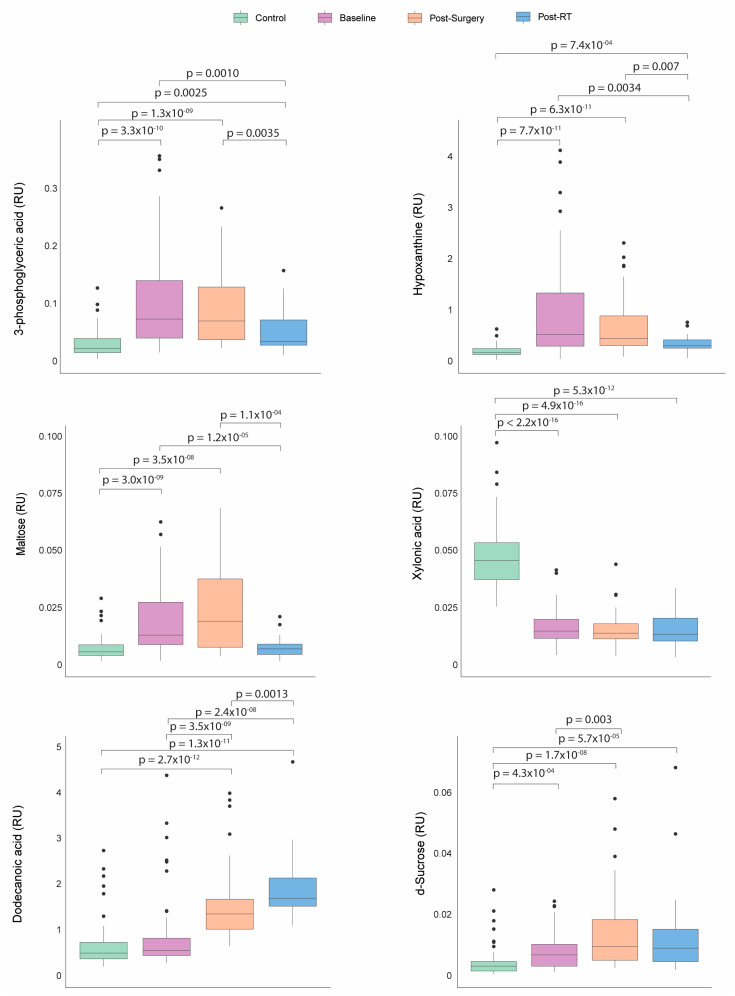
Dynamics of metabolic profile changes in breast cancer (BC) patients during different treatment courses. As treatment progressed, the metabolic profiles of the BC patients exhibited a trend towards normalization in the levels of 3-phosphoglyceric acid, hypoxanthine, maltose, and d-sucrose. Concurrently, the concentrations of dodecanoic acid increased while xylonic acid levels remained low. Statistical significance for comparisons was determined using the Mann–Whitney U test. RT: radiotherapy; RU: relative units.

Before any treatment, the patients with BC presented significantly higher plasma concentrations of hypoxanthine, 3-phosphoglyceric acid, maltose, and d-sucrose, along with lower concentrations of xylonic acid than the control group. Following the surgical intervention, sucrose and dodecanoic acid levels increased. After RT, the BC patients experienced a significant decrease in the levels of hypoxanthine, 3-phosphoglyceric acid, maltose, and sucrose, with a tendency towards normalization. Conversely, the dodecanoic acid concentrations remained significantly elevated compared to baseline and post-surgical levels (Figure 4).

## 4. Discussion

Our investigation has discerned a substantial number of metabolites showing altered concentrations in BC and undergoing modifications following surgery and RT. Using statistical models, we identified metabolites with the most relevant alterations, including hypoxanthine, 3-phosphoglyceric acid, maltose, sucrose, xylonic acid, and dodecanoic acid. The elevated plasma levels of hypoxanthine, 3-phosphoglyceric, and maltose in BC patients may signify the presence of metabolic disruptions linked to tumor growth and the tumor’s adaptation to its local environment. Despite these three molecules participating in distinct metabolic pathways, a unified interpretation can be achieved by considering their interconnections and shared metabolic shifts in cancer.

Hypoxanthine can originate through various deamination mechanisms during chronic inflammation [20,21] and potentially contribute to oncogenesis, given its essential role in synthesizing purine nucleotides, which are pivotal for metabolic regulation and cellular replication. The regulation of purine nucleotide synthesis pathways, encompassing both de novo synthesis and the salvage pathway, is imperative to fulfill the demand for nucleic acid precursors during cell proliferation. Indeed, disruptions in purine pools can impede cell proliferation and encourage apoptosis, particularly pertinent in tumor cells with flawed apoptosis-inducing pathways [22]. Hypoxanthine salvage significantly contributes to purine synthesis, with the observations of its depletion from extracellular media and its accumulation at intracellular levels under specific conditions implying its contribution to the ATP pool in these cells [23].

Emerging evidence has linked the dysregulation of purine metabolism to cancer, with heightened purine biosynthesis associated with the progression of various cancer types, including hepatocellular carcinoma, cholangiocarcinoma, glioblastoma, and lung cancer [24,25,26,27]. The metabolomic profiling of tumor tissues from lung cancer patients has uncovered increased levels of ribose-5-phosphate, indicative of accelerated purine synthesis required by highly proliferative cancer cells, along with the significant accumulations of hypoxanthine and xanthine [28,29]. Elevated plasma hypoxanthine concentrations have also been documented in breast cancer [30], ovarian cancer [31], and gastric cancer [32].

3-phosphoglyceric acid, an intermediate metabolite within the glycolytic pathway, is a critical component in ATP generation. In typical circumstances, 3-phosphoglyceric acid undergoes conversion into pyruvate, subsequently entering the mitochondria for complete oxidation in the Krebs cycle. Nevertheless, even with ample oxygen, heightened glycolytic activity persists in cancer, a phenomenon known as the Warburg effect [33]. This metabolic shift towards glycolysis may lead to the increased production of 3-phosphoglyceric acid, as tumor metabolism prioritizes the generation of intermediates suitable for macromolecular biosynthesis, thereby conferring a growth advantage. For instance, serine biosynthesis initiates with the oxidation of 3-phosphoglyceric acid into 3-phosphohydroxypyruvate and nicotinamide adenine dinucleotide catalyzed by phosphoglycerate dehydrogenase. The subsequent reductive amination of this ketone by phosphoserine yields 3-phosphoserine subsequently hydrolyzed into serine through the action of phosphoserine phosphatase [34]. Serine is a pivotal amino acid, playing a crucial role in supporting numerous anabolic processes, including synthesizing proteins, lipids, and nucleic acids [35,36,37]. Under conditions of serine deficiency, tumor cells can use hypoxanthine as a precursor for the synthesis of purines, so high plasma concentrations of 3-phosphoglycerate and hypoxanthine can greatly enhance the process of tumorigenesis [23].

Maltose is a disaccharide composed of two glucose molecules and is involved in the degradation of complex carbohydrates. The increased maltose levels in BC patients may indicate increased enzymatic activity of maltase, which breaks down complex carbohydrates into simpler sugars. Cancer cells often have a higher requirement for glucose as an energy source due to their altered metabolism. This increased degradation of complex carbohydrates may be an adaptation of the tumor to meet its energy and biosynthesis needs [38].

In summary, alterations in the plasma concentrations of hypoxanthine, 3-phosphoglycerate, and maltose in BC individuals indicate significant metabolic reprogramming within the tumor microenvironment. An augmented emphasis on nucleotide biosynthesis characterizes this reprogramming, heightened reliance on carbohydrates as an energy substrate, and a pronounced preference for glycolytic pathways. These metabolic adaptations have been closely linked to pivotal processes in cancer pathogenesis, including cell proliferation, enhanced survival, resistance to apoptosis, and heightened invasive potential.

One primary objective of the current investigation was to scrutinize alterations in the metabolomic profile of BC patients following surgical intervention and RT. The circulating metabolites with pro-oncogenic properties mentioned earlier tended to return to baseline following these therapeutic modalities. A previous study by our research group observed the partial normalization of certain metabolic pathways following RT [39,40]. However, it had two fundamental limitations: it focused on only 21 metabolites related to energy metabolism, and pre-RT blood samples were collected post-surgery, without pre-surgery samples, preventing us from assessing the effects of the surgical procedures. In contrast, the present study has allowed us to measure a broader range of metabolites in patients, taken after diagnosis but before the start of any treatment. This analysis provides new insights into metabolic reprogramming associated with primary therapies. Moreover, a marked increase in dodecanoic acid levels was observed. This compound (also termed lauric acid) is a medium-chain saturated fatty acid (12:0) present in the diet in vegetables and dairy products and not synthesized by the human body. The precise mechanisms responsible for the post-surgical and post-RT surge in plasma dodecanoic acid levels remain indiscernible from our findings. However, it is plausible that these changes may be attributed to alterations in dietary habits, lifestyle modifications, or perturbations in the gut microbiota. Regardless of the underlying cause, these shifts hold favorable implications from a metabolic standpoint, as dodecanoic acid has demonstrated anti-oncogenic properties. Prior research has indicated that dodecanoic acid can induce apoptosis by diminishing reduced glutathione availability and provoking oxidative stress in cancer cell lines such as Caco-2 and IEC-6 [41]. Subsequent investigations in colon cancer cells have suggested that the anticancer effects of dodecanoic acid may be partially mediated through the downregulation of the epidermal growth factor receptor, a pivotal player in apoptosis regulation and cancer cell survival [42]. Furthermore, dodecanoic acid has been observed to suppress the expression of oncogenic microRNAs in HepG2 and KB cells [43]. Significantly, studies have also found lower dodecanoic acid levels in the breast adipose tissue of BC patients when compared to healthy counterparts [44]. In addition, the pharmacological and dietary administration of this compound has yielded promising outcomes in cancer treatment [45,46,47]. Interestingly, our study showed higher post-RT dodecanoic acid concentrations in patients who had received adjuvant chemotherapy than in those who had not. Therefore, our results suggest that administering chemotherapy before radiation enhances therapeutic benefits by increasing dodecanoic acid levels in the bloodstream. These findings underscore the potential of dodecanoic acid in cancer treatment, offering optimism and encouragement in the fight against cancer. 

We have also found elevated sucrose levels after surgery. Surgical patients commonly develop hyperglycemia related to the hypermetabolic stress response, which increases glucose production and causes insulin resistance [48]. However, we find it difficult to believe these alterations persist a month after the procedure. As in the case of dodecanoic acid, this increase could be due to dietary changes or changes in the intestinal microbiota of these patients. In any case, this alteration is temporary and is corrected after RT.

Finally, our results show that the patients with BC had a lower concentration of xylonic acid in plasma than healthy volunteers, and this alteration persists throughout the treatments. To our knowledge, there is no prior information on the circulating levels of this compound in cancer. Xylonic acid is a sugar derived from xylose and synthesized by plants and microorganisms, but not by humans, and is used as an additive in the food industry. Some studies have reported that it has anti-inflammatory properties, inhibiting the synthesis of adhesins and the adhesion of macrophages, and can reduce inflammation-associated immune responses in some infectious diseases [49,50,51]. Its role in cancer is, at present, unknown.

While providing valuable insights, this study is subject to certain limitations that warrant consideration. Firstly, as a single-center study, the broader applicability of our findings may be influenced by factors such as ethnicity, environmental variations, and dietary habits. Consequently, caution should be exercised when attempting to generalize our results to the global population. Secondly, the sample size is limited, primarily due to the operational challenges posed by the COVID-19 pandemic. Logistical constraints during this unprecedented period hindered our ability to include a larger cohort of patients and collect a more extensive set of plasma samples. As a result, the findings cannot be considered totally conclusive and should be interpreted with the awareness of the study’s limitations in sample diversity and size. Finally, the study design does not allow us to definitively determine whether the observed effects following surgery are directly attributable to tumor removal or are a consequence of surgical trauma.

## 5. Conclusions

This preliminary study suggests that oncological treatments modify the metabolism of patients towards a favorable profile with a decrease in the pathways that favor cell proliferation and an increase in the plasma concentration of anticancer molecules. Specifically, the reduction in the pathways that favor cell proliferation, and the increase in protective metabolites, such as dodecanoic acid, could be linked to reduced resistance to cell death. Cancer cells that rely heavily on altered metabolism for survival might become more vulnerable to cell death mechanisms when these metabolic pathways are targeted or normalized. The knowledge acquired from our study and future research not only has the potential to enhance our comprehension of tumor cell behavior but also holds promise for the development of new avenues of research and discovery, such as therapy monitoring, the identification of novel therapeutic targets, and the integration of new blood-based biomarkers. Consequently, these discoveries may facilitate early and straightforward BC detection and advance the development of personalized treatment strategies.

## Figures and Tables

**Table 1:** Clinical and biochemical characteristics of the breast cancer patients and the control group.

	Control Group	BC Patients	*p*-Value
	(n = 49)	(n = 52)	
**Clinical characteristics**			
Age at diagnosis (years)	44.1 (14.9)	59.7 (12.4)	3.4 × 10^−7^
BMI	26.0 (4.7)	27.7 (5.5)	0.124
Smoking habit	18 (43.9)	10 (19.2)	0.019
Diabetes mellitus	3 (6.1)	7 (13.5)	0.368
Hypertension	5 (10.2)	17 (32.7)	0.013
Dyslipidemia	1 (2.0)	12 (23.1)	0.004
Premenopausal	-	14 (26.9)	
Perimenopausal	-	2 (3.8)	
Postmenopausal	-	36 (69.2)	
Use of oral contraceptives	-	15 (28.8)	-
Motherhood	-	42 (80.8)	-
Family cancer history	-	30 (57.7)	-
**Biochemical characteristics**			
Glucose (mmol/L)	4.7 (4.2–5.1)	5.4 (4.8–5.7)	4.1 × 10^−5^
Hemoglobin (g/dL)	13.7 (13.2–14.2)	13.5 (12.9–14.1)	0.301
Leukocytes (×10^9^/L)	6.6 (5.3–7.7)	6.6 (5.4–7.7)	0.846
Platelets (×10^9^/L)	245.7 (214.0–278.0)	254.2 (215.5–291.0)	0.341
Creatinine (mg/dL)	0.7 (0.6–0.8)	0.7 (0.7–0.7)	0.424
Total cholesterol (mmol/L)	5.1 (4.1–5.6)	5.4 (4.8–6.0)	0.026
HDL-cholesterol (mmol/L)	1.7 (1.4–2.0)	1.6 (1.3–1.7)	0.241
LDL-cholesterol (mmol/L)	2.9 (2.3–3.1)	3.2 (2.5–3.7)	0.085
VLDL-cholesterol (mmol/L)	0.4 (0.3–0.5)	0.7 (0.5–0.7)	5.2 × 10^−6^
Triglycerides (mmol/L)	1.0 (0.7–1.1)	1.5 (1.0–1.4)	1.4 × 10^−5^
GOT (µKat/L)	0.3 (0.3–0.4)	0.3 (0.3–0.4)	0.185
GPT (µKat/L)	0.3 (0.2–0.3)	0.3 (0.2–0.4)	0.064
GGT (µKat/L)	0.3 (0.1–0.3)	0.4 (0.2–0.4)	1.1 × 10^−6^
CCL2 (pg/mL)	77.7 (73.2–83.1)	78.8 (50.4–92.1)	0.063
IL-10 (ng/mL)	3.7 (2.2–4.1)	7.0 (3.1–6.3)	4.6 × 10^−4^
PON 1 concentration (pg/mL)	1.2 (0.2–1.5)	2.3 (0.2–2.8)	0.229
PON1 activity (U/L)	173.2 (147.9–204.6)	116.4 (37.9–177.1)	1.6 × 10^−4^

Results are shown as either n (percentage) or median (interquartile range). We conducted statistical analysis using the Mann–Whitney U test for quantitative variables, and the X-square test for qualitative variables. BMI: body mass index; CCL2: chemokine (C-C motif) ligand 2; GGT: γ-glutamyl transferase; GOT: glutamate-oxalacetate transaminase; GPT: glutamate-pyruvate transaminase; HDL: high-density lipoprotein; IL: interleukin; LDL: Low-density lipoprotein; PON1: paroxonase-1; VLDL: very low-density lipoprotein.

**Table 2:** Cancer characteristics.

	BC Patients
	(n = 52)
**Tumor size (TNM system)**	
T0	-
T1	33 (63.5)
T2	17 (32.7)
T3	2 (3.8)
T4	-
**Nodes (TNM system)**	
N0	35 (67.3)
N1	12 (23.1)
N2	4 (7.7)
N3	1 (1.9)
**Metastases (TNM system)**	
M0	52 (100)
M1	-
**Tumor histopathology**	
Ductal carcinoma	38 (42.2)
Lobular carcinoma	10 (11.1)
Other	4 (4.4)
Histological grade	
I	14 (26.9)
II	33 (63.5)
III	5 (9.6)
Positive estrogen receptors	97 (90–100)
Positive progesterone receptors	70 (11.7–95.0)
Positive HER2 in tumor biopsy	49 (94.2)
Ki67 antigen in tumor biopsy	22.5 (12.0–30.0)
**Tumor molecular classification**	
Luminal A	18 (34.6)
Luminal B	27 (51.9)
HER2 positive	2 (3.8)
Triple negative	5 (9.6)
Type of surgery	
Lumpectomy	40 (76.9)
Mastectomy	12 (23.1)
**Follow up**	
Alive	52 (100)

Results are shown as either n (percentage) or median (interquartile range). HER2: Human epidermal growth factor receptor 2.

## Data Availability

The raw data supporting the conclusions of this article will be made available by the authors upon request.

## Supplementary Materials

The following supporting information can be downloaded at: https://www.mdpi.com/article/10.3390/biomedicines12102196/s1, Table S1. Analytical standards; Table S2. Baseline plasma concentrations (relative units) of metabolites of healthy controls and patients with breast cancer (BC); Figure S1. Influence of age, smoking status, hypertension, and dyslipidemia on the concentrations of hypoxanthine, 3-phosphoglyceric acid, maltose, and xylonic acid in baseline BC patients and control group; Table S3. Plasma concentrations (relative units) of metabolites of healthy controls and post-surgery breast cancer (BC) patients; Figure S2. Effect of age, dyslipidemia, hypertension, and smoking status on the concentrations of d-sucrose, dodecanoic acid, and xylonic acid in post-surgery patients and the control group; Table S4. Plasma concentrations (relative units) of metabolites of healthy controls and post-radiotherapy breast cancer (BC) patients; Figure S3. Effect of age, dyslipidemia, hypertension, and smoking status on the concentrations of d-sucrose, dodecanoic acid, and xylonic acid in post-radiotherapy (post-RT) patients and the control group. Table S5. Comparison of post-radiotherapy plasma concentrations (relative units) of metabolites between patients receiving prior adjuvant chemotherapy (ACT) or not.
