# Supplementary material for: Preliminary Metabolomics Study Suggests Favorable Metabolic Changes in the Plasma of Breast Cancer Patients after Surgery and Adjuvant Treatment
Source: Biomedicines. 2024 Sep 26;12(10):2196. doi: 10.3390/biomedicines12102196 (PMC11505071; doi:10.3390/biomedicines12102196)
Supplement: Supplementary file 1 [file biomedicines-12-02196-s001.zip › biomedicines-3193389-supplementary.pdf]

Table S1. Analytical standards.

Set of standards (Sigma Aldrich)	
Pyruvic acid	a-ketoglutaric acid
Lactic acid	Arabitol
Glycolic acid	Glycerol-1-phosphate
3-hydroxybutyric acid	3-phosphoglyceric acid
Glycerol	Citric acid
Succinic acid	d-mannitol
Glyceric acid	Myo-inositol
Fumaric acid	Glucose-6-phosphate
Malic acid	d-sucrose
d-threitol	a-tocopherol
Threonic acid	
**Internal standards**	
Sigma Aldrich	
Succinic-d4 acid	d-glucose $^{13}C_6$
Myristic-d27	L-methionine-(carboxy-13C, methyl-d3)
Cambridge Isotope Laboratories	
Labeled amino acid mix standards	

Table S2. Baseline plasma concentrations (Relative Units) of metabolites of healthy controls and patients with breast cancer (BC).

	**Control** (n = 49)	**Baseline BC** (n = 52)	***p*- Value**
2-Hydroxybutyric acid	3.5 (2.0-3.4)	4.3 (2.7-5.5)	**0.029**
2-Hydroxyisobutyric acid	1.2(0.9-1.3)	1.4 (1.0-1.6)	**0.008**
2-Hydroxyisovaleric acid	4.8 (3.2-5.2)	5.9 (3.6-6.5)	0.091
2-keto-3-methylvaleric acid	1.2 (0.9-1.5)	1.1 (0.8-1.3)	**0.038**
3-hydroxybutyric acid	13.1 (5.1-18.6)	16.5 (4.7-22.7)	0.841
3-Hydroxyisovaleric acid	0.9 (0.6-1.1)	1.0 (0.7-1.2)	0.545
3-methyl-2-oxobutyric acid	0.6 (0.4-0.7)	0.6 (0.4-0.7)	0.379
3-Phosphoglyceric acid	0.03 (0.02-0.04)	0.03 (0.04-0.1)	**$3.3x10^{-10}$**
4-Hydroxybenzoic acid	0.12 (0.12-0.13)	0.12 (0.11-0.13)	0.284
4-hydroxyPhenyllactic acid	0.2 (0.1-0.2)	0.2 (0.1-0.2)	0.101
4-Hydroxyproline	1.6 (1.1-1.9)	1.5 (1.0-1.9)	0.836
a-ketoglutaric acid	0.2 (0.1-0.2)	0.2 (0.1-0.2)	0.739
a-tocopherol	0.3 (0.1-0.4)	0.2 (0.1-0.3)	0.372
Alanine	21.1 (17.7-24.2)	24.6 (20.9-28.5)	**0.001**
Benzoic acid	0.5 (0.3-0.7)	0.6 (0.4-0.7)	0.074
Citric acid	17.5 (13.9-19.3)	22.2 (15.6-27.8)	**0.012**
d-Arabinose	0.02 (0.01-0.02)	0.03 (0.02-0.02)	0.107
d-Arabitol	0.008 (0.007-0.01)	0.01 (0.009-0.01)	**$1.1x10^{-04}$**
d -Fructose	0.02 (0.01-0.02)	0.02 (0.01-0.03)	**0.038**
d-Galactitol	0.02 (0.02-0.03)	0.02 (0.01-0.02)	**0.032**
d-Mannonic acid	0.02 (0.02-0.03)	0.04 (0.02-0.04)	**0.006**
d-Sucrose	0.003 (0.002-0.005)	0.007 (0.003-0.01)	**$4.3x10^{-04}$**
d-Threitol	0.02 (0.02-0.02)	0.04 (0.02-0.03)	**$9.4x10^{-07}$**
d-Xylitol	0.001 (0.0009-0.001)	0.001 (0.001-0.002)	0.201
d-Xylose	0.007 (0.005-0.01)	0.008 (0.006-0.01)	0.081
DL-2-Hydroxyglutaric.acid	0.04 (0.03-0.05)	0.05 (0.03-0.05)	0.519
Dodecanoic acid	0.7 (0.4-0.7)	0.9 (0.5-0.8)	0.105
Erythronic acid	0.02 (0.02-0.03)	0.03 (0.02-0.04)	**$3.8x10^{-06}$**
Ethanolamine	0.2 (0.2-0.3)	0.2 (0.1-0.3)	0.749
Ethylmalonic acid	5.6 (4.4-6.3)	2.4 (1.7-2.9)	**$9.3x10^{-16}$**
Fumaric acid	0.13 (0.11-0.14)	0.13 (0.1-0.2)	0.887
Galacturonic acid	0.02 (0.01-0.02)	0.02 (0.01-0.02)	0.525
Glucose-6-phosphate	0.005 (0.004-0.007)	0.01 (0.007-0.01)	**$9.0x10^{-08}$**
Glutamic acid	3.9 (3.1-4.4)	6.2 (3.6-7.5)	**$1.1x10^{-04}$**
Glutamine	37.3 (28.2-44.4)	33.9 (26.3-39.3)	0.167
Glyceric acid	07 (0.5-0.8)	0.6 (0.4-0.6)	**0.003**
Glycerol	1.7 (1.2-2.1)	0.8 (0.5-1.0)	**$5.8x10^{-14}$**
Glycerol-1-phosphate	0.2 (0.1-0.2)	0.2 (0.1-0.3)	**0.039**
Glycine	6.5 (5.9-7.0)	6.6 (6.1-7.2)	0.383
Glycolic acid	0.2 (0.1-0.2)	0.2 (0.1-0.2)	**$1.6x10^{-06}$**
Hippuric acid	0.4 (0.1-0.4)	0.3 (0.1-0.4)	0.285

Hydrocinnamic acid	0.09 (0.04-0.1)	0.07 (0.03-0.1)	0.295
Hypoxanthine	0.2 (0.1-0.3)	1.1 (0.3-1.5)	**$7.7x10^{-11}$**
Indole-3-propanoic acid	0.1 (0.1-0.2)	0.2 (0.1-0.2)	0.313
Indolelactic acid	0.1 (0.1-0.2)	0.2 (0.1-0.2)	0.143
Isoleucine	4.5 (3.9-5.1)	4.6 (3.7-5.2)	0.932
Lactic acid	55.2 (42.8-62.4)	64.1 (54.5-73.1)	**0.003**
Leucine	8.9 (8.1-9.8)	9.1 (7.7-10.5)	0.981
Linoleic acid	3.1 (1.5-3.9)	3.4 (1.6-4.6)	0.927
Malic acid	0.06 (0.05-0.07)	0.08 (0.05-0.09)	**0.005**
Maltose	0.01 (0.004-0.01)	0.04 (0.01-0.04)	**$2.9x10^{-09}$**
Methionine	3.7 (3.5-3.8)	3.6 (3.3-3.9)	0.264
Myo Inositol	0.5 (0.4-0.5)	0.6 (0.5-0.7)	**$8.7x10^{-05}$**
Oleic acid	17.4 (7.8-24.7)	21.3 (11.0-24.4)	0.249
Ornithine	14.2 (9.8-16.7)	12.7 (9.7-14.8)	0.57
Oxoproline	25.7 (21.6-29.6)	25.4 (21.1-28.9)	0.82
Phenylalanine	6.3 (5.9-6.7)	6.4 (5.5-7.3)	0.711
Phosphoric acid	150.6 (66.2-209.1)	191.3 (155.9-233.8)	**0.013**
Proline	29.8 (21.5-33.8)	33.1 (23.5-41.8)	0.119
Pyruvic acid	4.9 (3.2-6.2)	5.3 (2.8-6.7)	0.783
Ribonic acid	0.003 (0.002-0.004)	0.004 (0.003-0.005)	**0.025**
Sedoheptulose	0.02 (0.01-0.02)	0.02 (0.01-0.02)	0.417
Serine	8.6 (7.4-9.5)	8.1 (6.2-9.4)	0.147
Succinic acid	0.06 (0.05-0.07)	0.08 (0.06-0.1)	**$2.8x10^{-04}$**
Taurine	0.5 (0.4-0.6)	1.2 (0.6-1.2)	**$4.2x10^{-08}$**
Tetradecanoic acid	1.9 (1.2-2.1)	1.9 (1.1-2.2)	0.539
Threonic acid	0.3 (0.2-0.3)	0.4 (0.2-0.4)	**$1.2x10^{-04}$**
Threonine	9.0 (7.3-10.1)	8.0 (6.7-9.0)	**0.035**
Uracil	0.02 (0.01-0.02)	0.02 (0.02-0.03)	0.114
Urea	30.7 (22.8-36.9)	35.5 (26.3-40.4)	0.057
Uric acid	11.2 (6.2-15.4)	13.3 (7.3-19.5)	0.267
Valine	16.9 (15.4-18.3)	17.5 (14.6-19.4)	0.666
Vanillylmandelic acid	0.006 (0.005-0.008)	0.007 (0.006-0.01)	**0.001**
Xylonic acid	0.05 (0.04-0.05)	0.02 (0.01-0.02)	**$6.5x10^{-17}$**

Values are provided as median (interquartile range) and compared with the Mann-Whitney U test. *P*- Values in bold indicate statistical significance ($p<0.05$).

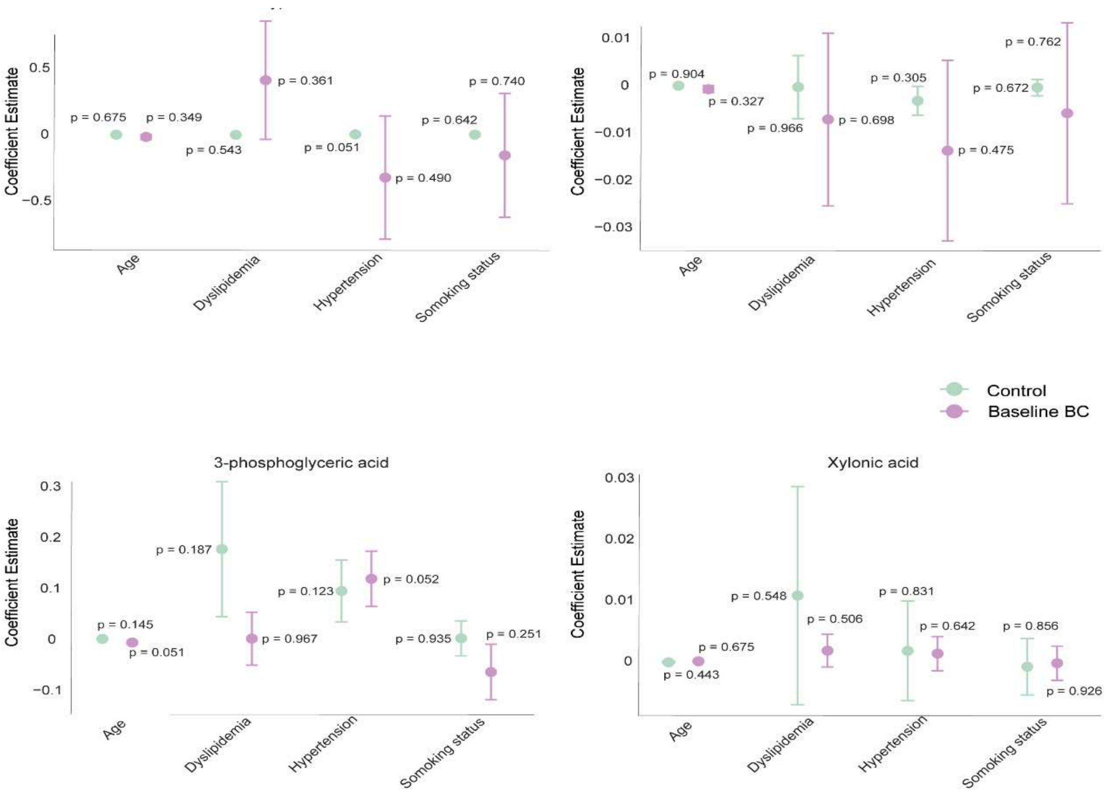

Figure S1. Influence of age, smoking status, hypertension, and dyslipidemia on the concentrations of hypoxanthine, 3-phosphoglyceric acid, maltose, and xylonic acid in baseline BC patients and control group. Each point represents the coefficient estimate from the multivariate regression analysis, with error bars indicating the standard error.

Table S3. Plasma concentrations (Relative Units) of metabolites of healthy controls and post-surgery breast cancer (BC) patients.

	**Control** (n = 49)	**Post-surgery** (n = 45)	***p*- Value**
2-Hydroxybutyric acid	3.5 (2.0-3.9)	3.8 (2.0-5.4)	0.261
2-Hydroxyisobutyric acid	1.2 (0.9-1.3)	1.7 (1.3-2.1)	**2.7X10^{-06}**
2-Hydroxyisovaleric acid	4.8 (3.2-5.2)	6.2 (4.1-7.1)	**0.001**
2-keto-3-methylvaleric acid	1.2 (0.9-1.5)	1.4 (1.2-1.7)	**0.042**
3-hydroxybutyric acid	13.1 (5.1-18.6)	8.2 (3.5-9.7)	**0.021**
3-Hydroxyisovaleric acid	0.9 (0.6-1.1)	1.3 (0.8-1.4)	**0.006**
3-methyl-2-oxobutyric acid	0.6 (0.4-0.7)	0.6 (0.5-0.6)	**0.650**
3-Phosphoglyceric acid	0.03 (0.02-0.04)	0.1 (0.04-0.1)	**1.3X10^{-09}**
4-Hydroxybenzoic acid	0.12 (0.12-0.13)	0.12 (0.11-0.12)	0.191
4-hydroxyPhenyllactic acid	0.2 (0.1-0.2)	0.2 (0.1-0.2)	**3.2X10^{-04}**
4-Hydroxyproline	1.6 (1.1-1.9)	1.4 (0.9-1.7)	0.157
a-ketoglutaric acid	0.2 (0.1-0.2)	0.1 (0.1-0.2)	0.895
Alanine	21.1 (17.7-24.2)	28.2 (25.6-31.5)	**4.9X10^{-08}**
a-tocopherol	0.3 (0.1-0.4)	0.3 (0.2-0.3)	0.537
Benzoic acid	0.5 (0.3-0.6)	0.8 (0.5-0.9)	**1.6X10^{-05}**
Citric acid	17.5 (14.0-19.3)	18.3 (12.9-22.9)	0.775
d-Arabinose	0.02 (0.01-0.02)	0.05 (0.02-0.04)	**2.9X10^{-07}**
d-Arabitol	0.01 (0.01-0.01)	0.01 (0.01-0.02)	**3.1X10^{-08}**
d-Fructose	0.02 (0.01-0.02)	0.08 (0.02-0.06)	**1.3X10^{-05}**
d-Galactitol	0.02 (0.02-0.02)	0.02 (0.02-0.02)	0.903
DL-2-Hydroxyglutaric acid	0.04 (0.03-0.05)	0.04 (0.03-0.05)	0.868
d-Mannonic acid	0.02 (0.02-0.03)	0.05 (0.03-0.05)	**1.3X10^{-07}**
Dodecanoic acid	0.7 (0.4-0.7)	1.6 (1.0-1.7)	**1.2X10^{-10}**
d-Sucrose	0.003 (0.002-0.005)	0.03 (0.005-0.02)	**1.6X10^{-08}**
d-Threitol	0.02 (0.02-0.02)	0.05 (0.02-0.03)	**6.3X10^{-11}**
d-Xylitol	0.001 (0.001-0.001)	0.002 (0.001-0.002)	**1.0X10^{-04}**
d-Xylose	0.007 (0.005-0.01)	0.02 (0.01-0.02)	**7.2X10^{-05}**
Erythronic acid	0.02 (0.01-0.03)	0.03 (0.02-0.03)	**3.9X10^{-05}**
Ethanolamine	0.2 (0.2-0.3)	0.2 (0.2-0.3)	0.756
Ethylmalonic acid	5.6 (4.4-6.3)	3.2 (2.5-4.1)	**1.2X10^{-10}**
Fumaric acid	0.13 (0.10-0.14)	0.2 (0.1-0.2)	0.026
Galacturonic acid	0.02 (0.01-0.02)	0.03 (0.02-0.03)	**7.8X10^{-05}**
Glucose-6-phosphate	0.005 (0.004-0.007)	0.01 (0.007-0.01)	**6.0X10^{-07}**
Glutamic acid	3.9 (3.1-4.4)	6.3 (4.3-8.2)	**4.4X10^{-05}**
Glutamine	37.3 (28.2-44.4)	31.0 (25.3-34.5)	**0.005**
Glyceric acid	0.7 (0.5-0.8)	0.5 (0.3-0.6)	**1.1X10^{-04}**
Glycerol	1.7 (1.2-2.1)	1.1 (0.9-1.3)	**1.2X10^{-07}**
Glycerol-1-phosphate	0.2 (0.1-0.2)	0.2 (0.1-0.2)	0.952
Glycine	6.5 (6.0-7.0)	7.2 (6.6-7.8)	**1.5X10^{-05}**
Glycolic acid	0.2 (0.2-0.3)	0.2 (0.2-0.3)	0.892
Hippuric acid	0.4 (0.1-0.4)	0.3 (0.1-0.4)	0.284

Hydrocinnamic acid	0.09 (0.04-0.1)	0.09 (0.04-0.1)	0.728
Hypoxanthine	0.2 (0.1-0.3)	1.3 (0.3-0.9)	**$1.1X10^{-09}$**
Indole-3-propanoic acid	0.1 (0.08-0.2)	0.2 (0.1-0.3)	**$1.8X10^{-04}$**
Indolelactic acid	0.1 (0.1-0.2)	0.2 (0.2-0.3)	**$4.5X10^{-06}$**
Isoleucine	4.5 (3.9-5.1)	5.4 (4.5-6.0)	**$4.3X10^{-04}$**
Lactic acid	55.2 (42.8-62.4)	87.6 (65.9-108.7)	**$1.1X10^{-08}$**
Leucine	8.9 (8.0-9.8)	10.0 (8.5-11.5)	**0.013**
Linoleic acid	3.1 (1.5-4.0)	2.3 (1.1-2.9)	**0.010**
Malic acid	0.06 (0.05-0.07)	0.08 (0.05-0.08)	**0.003**
Maltose	0.006 (0.004-0.01)	0.04 (0.01-0.05)	**$3.4X10^{-08}$**
Methionine	3.7 (3.5-3.8)	3.8 (3.3-4.0)	0.862
Myo Inositol	0.5 (0.4-0.5)	0.7 (0.6-0.9)	**$6.9X10^{-09}$**
Oleic acid	17.4 (7.8-24.7)	16.1 (7.0-20.8)	0.258
Ornithine	14.1 (9.8-16.7)	15.5 (11.4-18.7)	0.118
Oxoproline	25.7 (21.6-29.6)	29.3 (24.1-34.5)	**0.006**
Phenylalanine	6.3 (5.9-6.7)	6.7 (5.7-7.4)	0.612
Phosphoric acid	150.7 (66.2-209.1)	334.9 (272.5-374.4)	**$1.2X10^{-19}$**
Proline	29.8 (21.5-33.8)	33.8 (25.9-41.1)	**0.021**
Pyruvic acid	4.9 (3.2-6.2)	7.4 (4.8-10.2)	**$2.7X10^{-04}$**
Ribonic acid	0.003 (0.002-0.004)	0.004 (0.003-0.005)	**0.012**
Sedoheptulose	0.02 (0.01-0.02)	0.02 (0.02-0.02)	**$1.4X10^{-04}$**
Serine	8.6 (7.4-9.5)	8.6 (7.4-9.6)	0.527
Succinic acid	0.06 (0.05-0.06)	0.09 (0.05-0.1)	**0.001**
Taurine	0.5 (0.4-0.7)	1.3 (0.7-1.4)	**$1.1X10^{-08}$**
Tetradecanoic acid	2.0 (1.2-2.1)	2.1 (1.3-2.3)	0.201
Threonic acid	0.3 (0.2-0.3)	0.3 (0.3-0.4)	**$1.7X10^{-05}$**
Threonine	9.0 (7.3-10.1)	8.6 (7.0-9.8)	0.316
Uracil	0.02 (0.01-0.02)	0.03 (0.02-0.03)	**0.040**
Urea	30.7 (22.8-36.9)	46.6 (35.0-55.7)	**$1.6X10^{-07}$**
Uric acid	11.2 (6.2-15.4)	17.8 (10.6-23.0)	**$2.7X10^{-04}$**
Valine	16.9 (15.4-18.3)	19.3 (16.5-21.5)	**$2.0X10^{-04}$**
Vanillylmandelic acid	0.006 (0.005-0.008)	0.01 (0.008-0.01)	**$3.8X10^{-06}$**
Xylonic acid	0.05 (0.04-0.05)	0.02 (0.01-0.02)	**$4.9X10^{-16}$**

Values are provided as median (interquartile range) and compared with the Mann-Whitney U test. *P*- Values in bold indicate statistical significance ($p<0.05$).

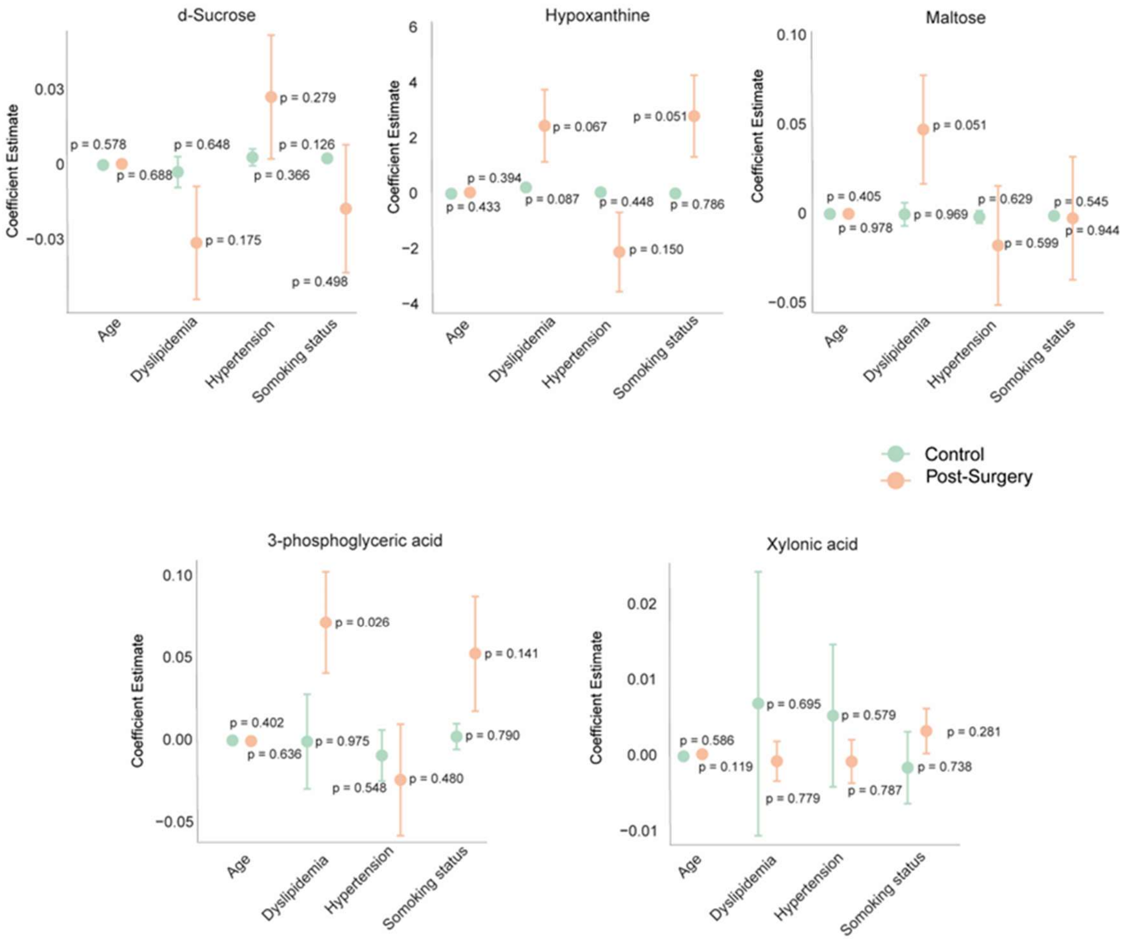

Figure S2. Effect of age, dyslipidemia, hypertension, and smoking status on the concentrations of d-sucrose, dodecanoic acid, and xylonic acid in post-surgery patients and the control group. Coefficient estimates from the multivariate regression analysis are displayed, with error bars representing the standard error. The analysis shows that these clinical and demographic variables do not significantly influence the concentrations of the metabolites studied.

Table S4. Plasma concentrations (Relative Units) of metabolites of healthy controls and post-radiotherapy breast cancer (BC) patients.

	**Control** (n = 49)	**Post-RT** (n = 26)	***p*-Value**
2-Hydroxybutyric acid	3.5 (2.0-3.9)	4.6 (3.0-6.2)	**0.005**
2-Hydroxyisobutyric acid	1.2 (0.9-1.3)	1.9 (1.3-2.4)	**5.0X10^{-06}**
2-Hydroxyisovaleric acid	4.8 (3.2-5.2)	5.2 (3.9-6.2)	**0.043**
2-keto-3-methylvaleric acid	1.2 (0.9-1.5)	1.4 (0.9-1.8)	0.311
3-hydroxybutyric acid	13.1 (5.2-18.6)	18.1 (5.5-17.6)	0.496
3-Hydroxyisovaleric acid	0.9 (0.6-1.1)	1.3 (0.9-1.5)	**0.005**
3-methyl-2-oxobutyric acid	0.6 (0.4-0.7)	0.76 (0.6-0.8)	**0.014**
3-Phosphoglyceric acid	0.03 (0.02-0.04)	0.05 (0.03-0.07)	**0.002**
4-Hydroxybenzoic acid	0.12 (0.12-0.13)	0.12 (0.11-0.12)	**0.046**
4-hydroxyPhenyllactic acid	0.2 (0.1-0.2)	0.2 (0.1-0.3)	**0.010**
4-Hydroxyproline	1.6 (1.1-1.9)	1.5 (0.9-1.9)	0.654
a-ketoglutaric acid	0.2 (0.1-0.2)	02 (0.1-0.2)	0.084
Alanine	21.1 (17.7-24.2)	26.7 (20.9-30.1)	**4.3X10^{-05}**
a-tocopherol	0.3 (0.1-0.4)	0.3 (0.2-0.4)	**0.049**
Benzoic acid	0.5 (0.3-0.6)	0.7 (0.4-0.9)	**0.030**
Citric acid	17.5 (13.9-19.3)	22.9 (17.9-25.6)	**0.001**
d-Arabinose	0.02 (0.01-0.02)	0.04 (0.02-0.04)	**0.006**
d-Arabitol	0.01 (0.01-0.01)	0.02 (0.01-0.02)	**1.2X10^{-07}**
d-Fructose	0.02 (0.01-0.02)	0.03 (0.01-0.03)	0.266
d-Galactitol	0.02 (0.02-0.03)	0.02 (0.02-0.02)	0.256
DL-2-Hydroxyglutaric acid	0.04 (0.03-0.05)	0.04 (0.03-0.05)	0.898
d-Mannonic acid	0.02 (0.02-0.03)	0.04 (0.02-0.04)	**1.5x10^{-04}**
Dodecanoic acid	0.7 (0.4-0.7)	2.2 (1.5-2.2)	**1.3X10^{-11}**
d-Sucrose	0.003 (0.002-0.005)	0.01 (0.004-0.02)	**5.7X10^{-05}**
d-Threitol	0.02 (0.02-0.02)	0.08 (0.02-0.03)	**9.8X10^{-09}**
d-Xylitol	0.001 (0.001-0.001)	0.02 (0.02-0.004)	**0.004**
d-Xylose	0.007 (0.005-0.01)	0.01 (0.01-0.02)	**0.005**
Erythronic acid	0.02 (0.02-0.03)	0.04 (0.02-0.04)	**7.6X10^{-05}**
Ethanolamine	0.2 (0.2-0.3)	0.2 (0.2-0.3)	0.867
Ethylmalonic acid	5.6 (4.4-6.3)	3.3 (2.3-4.1)	**3.4X10^{-08}**
Fumaric acid	0.1 (0.1-0.1)	0.2 (0.1-0.2)	**0.022**
Galacturonic acid	0.02 (0.02-0.02)	0.02 (0.02-0.03)	**0.011**
Glucose-6 -hosphate	0.007 (0.006-0.01)	0.005 (0.004-0.007)	**3.3X10^{-05}**
Glutamic acid	3.9 (3.1-4.4)	4.76 (2.73-6.81)	0.468
Glutamine	37.3 (28.2-44.4)	35.9 (26.2-40.5)	0.728
Glyceric acid	0.7 (0.5-0.8)	0.4 (0.3-0.4)	**7.4X10^{-09}**
Glycerol	1.7 (1.2-2.1)	1.1 (0.7-1.4)	**1.1X10^{-05}**
Glycerol-1-phosphate	0.2 (0.1-0.2)	0.2 (0.1-0.2)	0.442
Glycine	6.5 (5.9-6.9)	6.9 (6.5-7.3)	**0.014**
Glycolic acid	0.2 (0.1-0.2)	0.2 (0.2-0.3)	0.828
Hippuric acid	0.4 (0.1-0.4)	0.4 (0.1-0.4)	0.978

Hydrocinnamic acid	0.09 (0.04-0.1)	0.08 (0.04-0.1)	0.960
Hypoxanthine	0.2 (0.1-0.3)	0.7 (0.3-0.4)	**0.001**
Indole-3-propanoic acid	0.1 (0.08-0.2)	0.2 (0.1-0.2)	**0.006**
Indolelactic acid	0.2 (0.1-0.2)	0.2 (0.1-0.2)	**0.005**
Isoleucine	4.5 (3.9-5.1)	5.0 (4.3-5.8)	0.136
Lactic acid	55.2 (42.8-62.4)	69.9 (51.7-88.4)	0.034
Leucine	8.9 (8.1-9.8)	10.1 (8.6-11.0)	**0.023**
Linoleic acid	3.1 (1.5-3.9)	3.0 (1.4-3.0)	0.623
Malic acid	0.06 (0.05-0.07)	0.08 (0.06-0.08)	**0.018**
Maltose	0.006 (0.004-0.01)	0.008 (0.004-0.01)	0.442
Methionine	3.7 (3.5-3.3)	3.9 (3.6-4.2)	**0.070**
Myo Inositol	0.5 (0.4-0.5)	0.7 (0.5-0.8)	**$4.4X10^{-08}$**
Oleic acid	17.4 (7.8-24.7)	20.8 (8.5-23.3)	0.654
Ornithine	14.1 (9.8-16.7)	13.9 (9.5-16.9)	0.812
Oxoproline	25.7 (21.6-29.6)	22.3 (17.3-25.6)	**0.012**
Phenylalanine	6.3 (5.9-6.7)	6.9 (6.4-7.4)	**0.018**
Phosphoric acid	150.6 (66.2-209.1)	292.1 (211.2-360.7)	**$3.2X10^{-07}$**
Proline	29.8 (21.5-33.8)	31.8 (20.3-37.7)	0.344
Pyruvic acid	4.9 (3.2-6.2)	8.1 (5.5-10.6)	**$2.9X10^{-05}$**
Ribonic acid	0.003 (0.002-0.004)	0.003 (0.002-0.005)	0.605
Sedoheptulose	0.02 (0.01-0.02)	0.02 (0.01-0.02)	0.060
Serine	8.6 (7.4-9.5)	8.9 (7.5-10.2)	0.576
Succinic acid	0.06 (0.05-0.07)	0.07 (0.05-0.07)	**0.025**
Taurine	0.5 (0.4-0.6)	1.2 (0.5-1.5)	**0.003**
Tetradecanoic acid	1.9 (1.2-2.1)	3.3 (1.8-3.3)	**0.001**
Threonic acid	0.3 (0.2-0.3)	0.4 (0.3-0.4)	**$1.2X10^{-05}$**
Threonine	9.0 (7.3-10.1)	9.8 (7.5-10.3)	0.448
Uracil	0.02 (0.01-0.02)	0.02 (0.01-0.02)	0.730
Urea	30.7 (22.8-36.9)	46.3 (35.0-53.6)	**$1.1X10^{-06}$**
Uric acid	11.2 (6.2-15.4)	17.6 (12.2-19.7)	**0.002**
Valine	16.9 (15.4-18.3)	19.5 (17.4-21.2)	**$3.7X10^{-04}$**
Vanillylmandelic acid	0.006 (0.005-0.008)	0.01 (0.008-0.01)	**$1.9X10^{-07}$**
Xylonic acid	0.05 (0.04-0.05)	0.02 (0.01-0.02)	**$5.3X10^{-12}$**

RT: Radiotherapy. Values are provided as median (interquartile range) and compared with the Mann-Whitney U test. *P*- Values in bold indicate statistical significance ($p<0.05$).

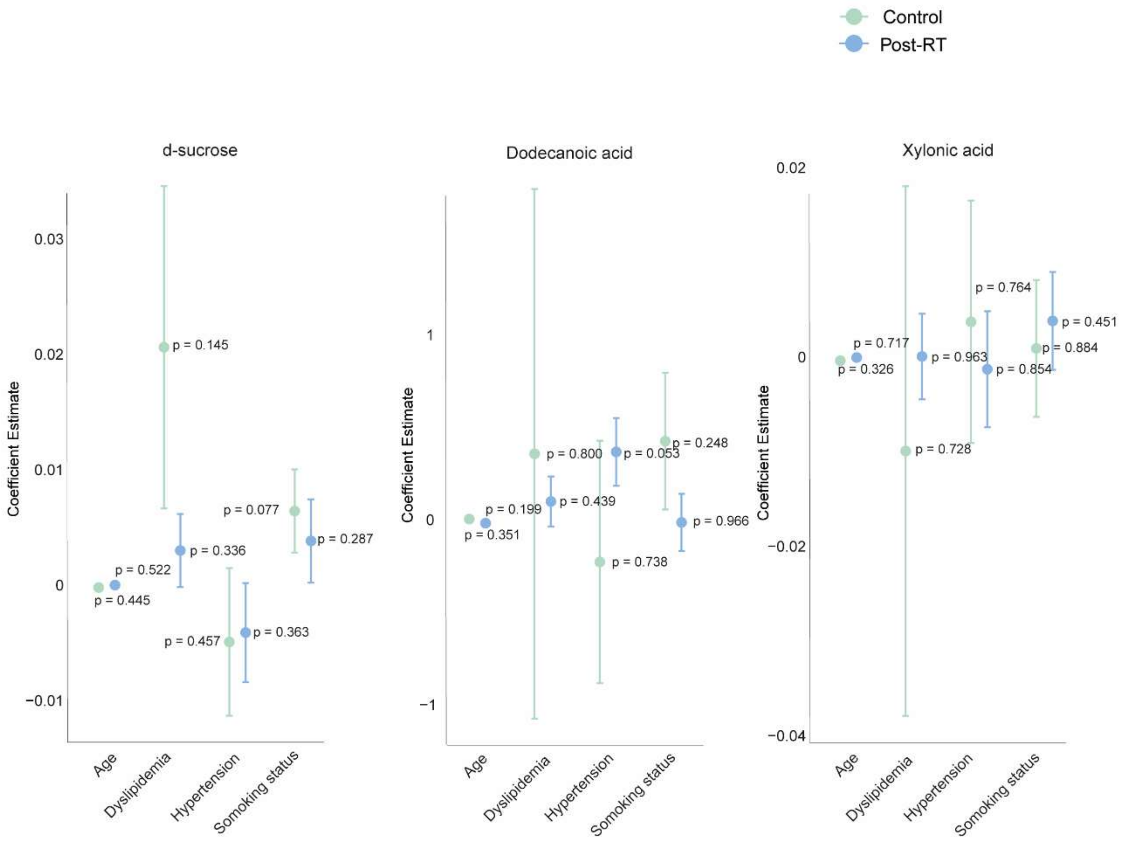

Figure S3. Effect of age, dyslipidemia, hypertension, and smoking status on the concentrations of d-sucrose, dodecanoic acid, and xylonic acid in post-radiotherapy (post-RT) patients and the control group. Coefficient estimates from the multivariate regression analysis are displayed, with error bars representing the standard error. The analysis shows that these clinical and demographic variables do not significantly influence the concentrations of the metabolites studied.

Table S5. Comparison of post-radiotherapy plasma concentrations (Relative Units) of metabolites between patients receiving prior adjuvant chemotherapy (ACT) or not.

	**ACT** (n = 11)	**no ACT** (n = 15)	***p*- Value**
2-hydroxybutyric acid	3.3 (2.8-4.2)	3.9 (3.6-7.0)	0.058
2-hydroxyisobutyric acid	1.6 (1.3-1.9)	1.8 (1.3-2.4)	0.287
2-hydroxyisovaleric acid	4.5 (3.2-5.7)	5.5 (4.8-6.9)	0.052
2-keto-3-methylvaleric acid	1.0 (0.9-1.5)	1.6 (1.1-1.8)	0.126
3-hydroxybutyric acid	11.1 (4.7-19.9)	8.8 (7.1-11.6)	0.551
3-hydroxyisovaleric acid	1.0 (0.8-1.5)	1.3 (0.9-1.5)	0.421
3-methyl-2-oxobutyric acid	0.6 (0.5-0.7)	0.8 (0.6-0.8)	**0.014**
3-phosphoglyceric acid	0.04 (0.03-0.07)	0.03 (0.03-0.08)	0.959
4-hydroxybenzoic acid	0.12 (0.12-0.13)	0.12 (0.11- 0.13)	0.567
4-hydroxyphenyllactic acid	0.2 (0.1-0.2)	0.2 (0.1-0.3)	0.364
4-hydroxyproline	1.9 (1.2-2.4)	1.2 (0.9-1.5)	0.092
α-ketoglutaric acid	0.16 (0.14-0.2)	0.14 (0.12-0.2)	0.659
α-tocopherol	0.3 (0.2-0.4)	0.3 (0.3-0.4)	0.484
Alanine	29.2 (24.8-30.2)	28.4 (20.6-30.7)	0.517
Benzoic acid	0.6 (0.5-0.8)	0.6 (0.4-0.9)	0.755
Citric acid	23.6 (20.7-29.7)	20.6 (15.7-23.6)	0.082
d-arabinose	0.02 (0.01-0.03)	0.03 (0.02-0.05)	0.091
d-arabitol	0.02 (0.01-0.02)	0.01 (0.01-0.02)	0.498
d-fructose	0.02 (0.01-0.02)	0.02 (0.01-0.04)	0.716
d-galactitol	0.02 (0.02-0.02)	0.02 (0.02-0.02)	0.754
d-mannonic acid	0.04 (0.02-0.05)	0.03 (0.03-0.04)	0.835
d-sucrose	0.002 (0.001- 0.01)	0.01 (0.01-0.02)	0.230
d-threitol	0.02 (0.02-0.03)	0.03 (0.02-0.04)	0.297
d-xylitol	0.002 (0.001-0.002)	0.002 (0.001-0.002)	0.441
d-xylose	0.02 (0.01-0.03)	0.007 (0.005-0.008)	**0.002**
DL-2-hydroxyglutaric acid	0.04 (0.04-0.06)	0.04 (0.04-0.05)	0.515
Dodecanoic acid	2.4 (2.2-4.8)	1.6 (1.3-1.7)	**$6.4x10^{-05}$**
Erythronic acid	0.03 (0.02-0.05)	0.03 (0.02-0.04)	0.736
Ethanolamine	0.2 (0.2-0.3)	0.2 (0.2-0.3)	0.391
Ethylmalonic acid	4.2 (3.6-4.6)	2.8 (2.3-3.4)	0.031
Fumaric acid	0.1 (0.1-0.2)	0.1 (0.1-0.2)	0.421
Galacturonic acid	0.02 (0.02-0.03)	0.02 (0.02-0.03)	0.855
Glucose 6-phosphate	0.007 (0.005-0.008)	0.008 (0.007-0.01)	0.219
Glutamic acid	4.2 (2.3-7.4)	3.7 (3.2-6.3)	0.736
Glutamine	35.8 (31.3-40.3)	36.5 (24.4-40.1)	0.421
Glyceric acid	0.4 (0.3-0.4)	0.4 (0.3-0.5)	0.815
Glycerol	1.3 (0.9-1.6)	0.8 (0.8-1.1)	0.186
Glycerol-1-phosphate	0.2 (0.2-0.3)	0.1 (0.1-0.2)	0.008
Glycine	7.1 (6.4-7.5)	6.8 (6.4-7.1)	0.364
Glycolic acid	0.2 (0.2-0.3)	0.2 (0.2-0.3)	0.622
Hippuric acid	0.2 (0.1-0.4)	0.3 (0.2-0.4)	0.586

Hydrocinnamic acid	0.05 (0.01-0.07)	0.07 (0.05-0.1)	0.073
Hypoxanthine	0.3 (0.2-0.4)	0.3 (0.3-0.5)	0.243
Indole-3-propanoic acid	0.2 (0.1-0.2)	0.2 (0.1-0.2)	0.659
Indolelactic acid	0.2 (0.1-0.2)	0.2 (0.1-0.2)	0.938
Isoleucine	4.4 (4.1-5.9)	4.7 (4.4-6.0)	0.421
Lactic acid	50.8 (45.8-64.2)	64.0 (57.9-97.6)	**0.036**
Leucine	8.5 (8.1-11.6)	9.9 (8.9-11.5)	0.243
Linoleic acid	3.9 (2.6-4.8)	1.7 (1.3-2.6)	**0.010**
Malic acid	0.07 (0.06-0.08)	0.06 (0.06-0.09)	0.917
Maltose	0.006 (0.003-0.01)	0.01 (0.008-0.01)	0.051
Methionine	3.8 (3.7-4.1)	3.6 (3.5-4.2)	0.364
Myo-inositol	0.6 (0.5-0.7)	0.7 (0.5-0.8)	0.337
Oleic acid	19.2 (14.0-37.4)	11.9 (8.1-20.5)	0.102
Ornithine	10.9 (9.3-13.2)	12.4 (11.1-18.7)	0.337
Oxoproline	22.0 (15.8-23.6)	22.5 (18.9-26.9)	0.312
Phenylalanine	6.4 (6.1-7.4)	6.9 (6.4-8.3)	0.392
Phosphoric acid	235.6 (195.6-358.0)	291.4 (225.3-384.5)	0.551
Proline	30.6 (23.9-36.1)	37.5 (20.5-42.1)	0.337
Pyruvic acid	5.8 (4.4-8.9)	8.9 (5.8-11.6)	0.092
Ribonic acid	0.004 (0.003-0.006)	0.003 (0.002-0.005)	0.618
Sedoheptulose	0.02 (0.01-0.02)	0.02 (0.02-0.03)	0.405
Serine	8.9 (8.8-10.7)	8.1 (6.8-10.1)	0.169
Succinic acid	0.07 (0.07-0.1)	0.06 (0.05-0.07)	0.119
Taurine	0.5 (0.5-0.8)	0.9 (0.6-1.0)	0.082
Tetradecanoic acid	5.2 (3.0-5.6)	1.8 (1.6-2.3)	**1.86x10^{-04}**
Threonic acid	0.3 (0.3-0.4)	0.4 (0.3-0.5)	0.586
Threonine	10.3 (9.4-12.7)	8.3 (7.3-9.7)	0.102
Uracil	0.02 (0.01-0.02)	0.02 (0.01-0.02)	0.404
Urea	34.7 (32.9-50.8)	47.3 (40.9-61.6)	**0.036**
Uric acid	17.1 (14.3-23.3)	13.6 (9.7-19.3)	0.126
Valine	17.4 (16.2-22.3)	20.2 (18.2-21.1)	0.186
Vanillylmandelic acid	0.009 (0.007-0.01)	0.01 (0.009-0.01)	0.261
Xylonic acid	0.02 (0.01-0.02)	0.01 (0.01-0.02)	0.515

Values are provided as median (interquartile range) and compared with the Mann-Whitney U test. *P*- Values in bold indicate statistical significance ($p<0.05$).
